# Cellular markers of mule duck livers after force-feeding

**DOI:** 10.1016/j.psj.2020.03.048

**Published:** 2020-04-25

**Authors:** Bara Lo, Nathalie Marty-Gasset, Hélène Manse, Carole Bannelier, Céline Bravo, Renaud Domitile, Hervé Rémignon

**Affiliations:** ∗ GenPhySE, Université de Toulouse, INRAE, ENVT, 31326 Castanet Tolosan, France; † IDENA, Sautron 44880, France

**Keywords:** *Foie gras*, hypoxia, oxidative stress, cell death, mule duck

## Abstract

The “*Foie gras*” or fatty liver is the result of hepatic steatosis from nutritional origin and induced by the force-feeding of palmipeds. Despite identical rearing and force-feeding conditions of ducks from the same breed, different liver weights, within a range of 500 to more than 700 g, are generally observed at the time of evisceration. To better understand the determinism of this large variability in fatty liver weights, the activity of various metabolic pathways has been explored in 4 groups of steatotic livers differing by their weights. Different analyses were performed using biochemical assays on metabolites as well as ELISA tests or enzyme activity assays. The result showed that an increase in the final liver weight is always associated with a hypoxic response and even a severe hypoxia observed in livers with the highest weights (more than 650 g). This is also combined with a rise in the cellular oxidative stress level. In addition, for the heaviest livers (more than 700 g), signs of cell death by apoptosis were also observed, while others programmed cell death pathways, such as ferroptosis or necroptosis, seemed to be nonactive.

## Introduction

The “*Foie gras”* is one of the most famous specialties of the French gastronomy. Then, France supplies about 80% of the “*Foie gras*” produced each year in the world, and 90% of the French population is a consumer of this product (CIFOG, 2018).

The “*Foie gras”* comes from the fatty hypertrophy of hepatocytes induced by the force-feeding of ducks with corn during 10 to 12 D. In ducks, at the end of this period of force-feeding, the liver must have a weight greater than 300 g to be legally called “*Foie gras”* (UE, 2008).

During the force-feeding period, ducks are fed twice a day with corn, usually in the form of a moistened flour. The corn contains a large quantity of starch (about 64%, Souci et al., 2000) which is transformed into fatty acids by the liver through the process of *de novo* lipogenesis. In addition, reabsorption of circulating lipids allows the liver to become the main organ of fat storage during this process (Théron, 2011). The imbalance between the neosynthesis and the export of lipids finally results in the accumulation of those lipids in hepatocytes as observed in hepatic steatosis (Hermier et al., 2003). In birds, this storage ability of lipids in the liver is natural and reversible (Babilé et al., 1996, Babilé et al., 1998). In palmipeds, food-induced hepatic steatosis is totally regulated by force-feeding because when it stopped, the animal recovers its initial status in only a few weeks (Théron, 2011). Based on rearing and force-feeding conditions, fatty liver of ducks can weigh from 300 to more than 800 g (Théron, 2011, Bonnefont et al., 2014, Bonnefont et al., 2019, Rémignon et al., 2018).

At the end of the force-feeding period, several modifications of various metabolic activities of the liver have been observed. For example, there is a huge increase in protein and lipid metabolisms but also in oxidative stress mechanisms and even cell death by apoptosis (Babilé et al., 1998, Bénard and Labie, 1998, Théron, 2011, Bonnefont et al., 2014, Rémignon et al., 2018). However, all the cellular mechanisms induced by force-feeding are not completely known and the presence of hepatic cell deaths by apoptosis was very recently described only in livers with a weight greater than 700 g (Rémignon et al., 2018).

According to Gautheron (2017), necroptosis is a form of cell death that shares the molecular machinery of the extrinsic apoptotic pathways with symptoms of necrosis. In humans, necroptosis could contribute to the development of nonalcoholic hepatic steatosis (**NASH**).

According to Xie et al. (2016), ferroptosis is an iron-dependent and reactive oxygen species (ROS)–dependent form of programmed cell death. It is distinct from other forms of programmed cell death at morphological, biochemical, and genetic levels. Recently, Tsurusaki et al. (2019) showed further evidence to suggest the involvement of ferroptosis in patients with NASH.

Owing to the excessive energy supply during the force-feeding period, the development of hepatic steatosis consumes a high quantity of oxygen associated with an increase in the number of ROS. The high level of ROS is a sign of an upward trend in oxidative stress. Machado and Diehl (2016) have demonstrated that the oxidative stress induced by the abnormal accumulation of lipids must be considered as an important initiation factor of NASH.

A reduction in the available amount of oxygen results in tissue hypoxia as described by Gradwell (2006). Suzuki et al. (2014) reported that a severe liver hypoxia is observed and is due to disrupted hepatic oxygen homeostasis caused by the altered hepatic metabolism and tissue remodeling in fatty liver disease.

The aim of the present study is to monitor different cellular markers in livers of different weights at the end of a force-feeding period. The main hypothesis to be tested is that during the force-feeding period, the very significant growth in the weight of the liver leads to a physical obstruction which, in combination with an increase in blood lipemia, induces discomfort in the microcirculation of blood. Then, the major consequence could be the development of a hepatic hypoxia caused by the decrease of oxygen partial pressure in the liver. In addition, this hypoxia combined with oxidative stress and other metabolic disorders could induce the activation of a programmed cell death pathway. Finally, depending on the weight of the liver, this cell death could be supported by apoptosis, necroptosis, or ferroptosis pathways.

## Materials and methods

### Animals and Samples

A flock of about 1,000 male mule ducks (*Caïrina moschata x Anas platyrhynchos*) was reared for 12 wk in accordance with standard commercial rules as described by Bonnefont et al. (2019). Then, birds were force-fed for 20 meals (twice a day during 10 D), in accordance with a standard force-feeding program based on moistened corn flour. At the beginning of the force-feeding period, ducks received a quantity of 230 g/meal which was progressively increased to a final value of 480 g for the last meal. As a whole, during the force-feeding period, ducks ingested an average quantity of 8.834 kg of feed.

Approximately 11 h after the last meal, the birds were slaughtered in a commercial slaughter house. Ducks first underwent electronarcosis and then bleeding, scalding, and plucking before liver evisceration (around 20 min post-mortem). Livers were automatically weighed, and 150 of them were randomly harvested to create 4 experimental groups composed of 40 livers weighing between 550 g and 599 g, 40 between 600 g and 649 g, 40 between 650 g and 700 g, and finally 30 with a weight greater than 700 g. From all those livers, 50 g was collected from the median lobe and directly frozen in liquid nitrogen before storage at −80°C.

The rest of the livers were cooled to 4°C on the processing line before the measurement of the color as per the CIELab system (L∗,a∗, b∗) performed on 3 different points located on the large lobe of the liver with a chromameter (CR 300 Minolta, Osaka, Japan). Then, near-infrared spectroscopy measurements were collected on 6 independent points of the surface of the 150 livers using a spectrometer (Labspec 5000 Pro, ASD Inc., Boulder, CO) to predict the gross biochemical characteristics of livers.

### Biochemical Analyses

For all the 150 samples, the gross biochemical (i.e., dry matter [**DM**], lipids, total nitrogen) contents were determined from near-infrared spectroscopy spectra in accordance with the method described by Marie-Etancelin et al. (2014). Forty-eight samples (12/groups) were then selected for being representatives of the whole 150 samples and further analyzed for several markers of biochemistry of the hepatocytes. The total amount of protein was determined by the following formula: (% Proteins = % total nitrogen × 6.25).

All the biochemical determinations were done in triplicate.

### Reduced Glutathione-to-Total Glutathione Ratio Analysis

The reduced glutathione (**GSH**)-to-total glutathione (**GT**) ratio (GSH/GT) was determined in accordance with the protocol described by the manufacturer (Catalog #: K264, BioVision Inc., CA). Briefly, comminuted livers were homogenized in ice-cold GSH assay buffer. Then, 60% of the homogenate was added to a prechilled centrifuge tube containing perchloric acid (6N) to precipitate protein. After collecting the supernatant and discarding the protein pellet, potassium hydroxide (**KOH**, 6N) was added to precipitate perchloric acid and neutralize the samples. Those mixtures were centrifuged, and each supernatant was taken for the assay.

For the GSH detection, the samples were diluted with the assay buffer. For the GT detection, a reducing agent was added to the wells, mixed, and incubated at room temperature. Finally, o-phthalaldehyde was added to all samples before incubation at room temperature for 40 min. The fluorescence intensity was then measured using a microplate reader CLARIOstar Plus (BMG LABTECH GmbH, Germany) at Ex/Em of 340/450 nm, respectively.

### Lipids Oxidation Assay

Lipids oxidation assay was performed with thiobarbituric acid reactive substances (**TBARS**) in accordance with the method of Lynch and Frei (1993). In an acidic medium, malondialdehyde (**MDA**) reacts with thiobarbituric acid (**TBA**) to give a pink-colored complex that displays maximum absorbence at 535 nm. The results are calculated from a calibration curve obtained with tetraethoxypropane that is transformed into MDA during heating and reacts with TBA. The results were expressed in mmol of MDA per g of fresh tissue.

### ELISA Tests

Hypoxia-inducible factor 1 alpha (**HIF1α**), hypoxia-inducible factor 2 alpha (**HIF2α**), glutathione peroxidase 4 (**GPX4**), receptor-interacting serine/threonine-protein kinase 3 (**RIPK3**), and serine/threonine-protein phosphatase (**PGAM5**, *[Phosphoglycerate mutase family member 5]*, mitochondrial) contents were determined with ELISA tests by using assay kits from MyBioSouce (San Diego, CA) and in accordance with the manufacturer's protocol. Briefly, 250 mg of each comminuted livers was homogenized in PBS buffer. Soluble proteins were collected in the supernatant after centrifugation at 10,000 g for 5 min. Protein concentration in the supernatant was then determined as per Bradford's test (Sigma-Aldrich, St Louis, MO). For each determination, the assay was performed with 300 μg of total proteins. Results are expressed in pg of proteins per g of fresh tissue except for GPX4 expressed in U/pg.

### Cathepsins Activities Assays

Measurements of cathepsins B, D, and L activities were performed using assay kits in accordance with the protocol described by the manufacturer (KA0766, KA0767, and KA0770, respectively, Abnova, Taiwan). Results are expressed in ratio of enzyme unit (U) per g of fresh tissue.

### Caspases Activities Assays

Measurements of caspases 3 + 7, 8, and 9 activities were performed using the respective caspase/Glo assay kits from Promega (Madison, WI) in accordance with the protocols described by the manufacturer. Results are expressed in ratio of enzyme unit (U) per g of fresh tissue.

### Statistical Analysis

Statistical analyses were performed using the SAS software (version 9.4 of the SAS System for Windows) and the R software (version 3.5.3). Before each analysis, data which did not validate the assumptions for parametric tests were standardized with logarithm transformation. Then, ANalyses Of VAriance were performed with the General Linear Model (Proc GLM) completed with the Student-Newman-Keuls’ post hoc test to compare the means of each groups. Simple correlations (Proc Corr) were determined in accordance with the method of Pearson. Finally, a canonical discriminant analysis (Proc Candisc) was performed to observe all the data (variables and observations) as a whole.

## Results and discussion

For the whole flock of about 1,000 slaughtered male mule ducks, the average liver weight was 580 g, while the average live body weight was 6.5 kg. As expected (Table 1), liver weights in the 4 studied groups were largely different (*P* < 0.001). On the contrary, no significant differences (*P* > 0.05) were observed for DM and total lipid contents and for color parameters between the 4 studied groups. However, the total protein contents from the 2 groups with the lowest weights (550–599 g and 600–649 g) were significantly higher (*P* < 0.001) than those from the intermediate (650–699 g) or heaviest (>700 g) liver weight groups.

**Table 1:** Liver weights (LW), gross chemical composition, and color parameters of livers from the different studied groups (n = 12 birds/groups).

Parameters	550 – 599 g	600 – 649 g	650 – 699 g	>700 g	*P*. value
LW (g)	576 ± 17^a^	626 ± 16^b^	676 ± 14^c^	767 ± 36^d^	∗∗∗
Lipids (% of dry matter)	57.1 ± 0.9	57.2 ± 1.7	57.4 ± 1.1	57.9 ± 1.2	NS
Dry matter (% of raw weight)	70.2 ± 0.9	70.4 ± 1.4	70.6 ± 1	70.9 ± 1.4	NS
Proteins (% of dry matter)	7.03 ± 0.43^a^	6.93 ± 0.44^a^	6.47 ± 0.43^b^	6 ± 0.43^c^	∗∗∗
L∗	71.4 ± 1.4	71.4 ± 1.9	71.4 ± 1.6	72.4 ± 1.5	NS
a∗	9.34 ± 0.94	9.32 ± 1.6	9.52 ± 1.43	9.01 ± 0.93	NS
b∗	29.06 ± 1.61	29.67 ± 2.13	28.8 ± 1.74	28.33 ± 1.64	NS

Values are means ± SD. Within a line, values with different superscripts differ significantly (∗*P* ≤ 0.05, ∗∗*P* ≤ 0.01, ∗∗∗*P* ≤ 0.001, and NS: not significant).

### Oxidative Stress

In the present study, a significant (Table 2, *P* < 0.001) decrease in the value of the GSH/GT ratio was observed when the livers weighed more than 650 g. The results also showed a nonsignificant difference between the 2 lowest and the 2 highest groups of liver weights for the value of the GSH/GT ratio (Table 2). Nevertheless, a significant difference was observed between livers with a weight lower than 650 g and livers weighing more than 650 g (*P* < 0.001).

**Table 2:** Quantity of metabolic marker involved in different metabolic pathways (n = 12 birds/groups).

Parameters		550 – 599 g	600 – 649 g	650 – 699 g	>700 g	*P*-value
Oxydative stress	GSH/GT ratio	0.18 ± 0.01^a^	0.19 ± 0.03^a^	0.13 ± 0.02^b^	0.13 ± 0.03^b^	∗∗∗
	TBARS (mmol MDA/g of fresh tissue)	4.12 ± 1.11	3.66 ± 0.83	4.1 ± 1.22	3.8 ± 0.8	NS
Hypoxia	HIF1α (pg/g of fresh tissue)	148 ± 25^a^	115 ± 26^b^	89 ± 38^c^	88 ± 34 ^c^	∗∗∗
	HIF2α (pg/g of fresh tissue)	46 ± 17.6^a,b^	35.8 ± 11.3^b^	64.7 ± 22.7^a^	53.7 ± 21.8^a,b^	∗∗
Necroptosis	RIPK3 (pg/g of fresh tissue)	2,676 ± 793	2,303 ± 820	2,217 ± 1,179	2,337 ± 1,346	NS
	PGAM5 (pg/g of fresh tissue)	2,980 ± 760	2,457 ± 835	2,557 ± 995	2,984 ± 1,846	NS
Ferroptosis	GPX4 (U/g of fresh tissue)	107 ± 30	86 ± 20	79 ± 50	71 ± 38	NS
Apoptosis	CASP3+7 (ratio/g of fresh tissue)	1,368 ± 627^a^	1,327 ± 433^a^	1,838 ± 2,222^a^	3,756 ± 3,628^b^	∗
	CASP8 (ratio/g of fresh tissue)	23,918 ± 6,579	23,630 ± 5,314	26,197 ± 16,156	39,232 ± 27,616	NS
	CASP9 (ratio/g of fresh tissue)	29,283 ± 6,376	28,761 ± 4,833	30,706 ± 14,616	41,455 ± 24,376	NS
	CathL (ratio/g of fresh tissue)	80.7 ± 18.6	77.3 ± 12	74.5 ± 15.7	71.1 ± 19.8	NS
	CathD (ratio/g of fresh tissue)	56.5 ± 5.9	53.7 ± 3.3	50.5 ± 7.4	51.2 ± 5.8	NS
	CathB (ratio/g of fresh tissue)	73 ± 6^a^	70.6 ± 5.7^a^	62.3 ± 9.4^b^	63 ± 7.3^b^	∗∗∗

Values are means ± SD. Within a line, values with different superscripts differ significantly (∗*P* ≤ 0.05, ∗∗*P* ≤ 0.01, ∗∗∗*P* ≤ 0.001).

Abbreviation: NS, not significant.

Another indicator for measuring the oxidative stress level in a tissue is the level of lipid peroxidation (Ghani et al., 2017) as assessed by TBARS values according to Lynch and Frei (1993). In the present study, the results did not show any significant differences between the 4 studied groups for TBARS values.

### Hypoxia

HIF1α and HIF2α are, respectively, markers of light and severe hypoxia as reported by Rankin et al. (2009). The results reported in Table 2 showed that the concentration of HIF2α in livers was relatively variable between the 4 studied groups. The lowest values were found in the livers weighing between 600 and 649 g, while the highest value was recorded in the livers weighing between 650 and 699 g. On the contrary, the concentration of HIF1α decreased when the liver weights increased to 650 g (*P* < 0.001).

### Cell Death Pathways

In the present study, no significant differences between the 4 studied groups for the activity of initiator caspases 8 and 9 were reported (Table 2). On the contrary, the activity of caspases 3 + 7 was found to be higher (*P* < 0.05) in livers with a weight more than 700 g than in those with the lowest weights. However, a very high variability existed in those measurements, especially for the groups with the highest weights (coefficients of variation = 121 and 96% in 650–699 and > 700 g groups, respectively). This high variability explains why a nonsignificant difference was reported between the different groups of liver weights but also reveals a complexity in the interpretation of the results and finally shows that a liver with a high weight will not inevitably present that sign of activation of cell death by apoptosis.

As reported in Table 2, the activities of cathepsins varied based on groups and the types of cathepsins. For the activities of the isoforms D and L, no significant differences were observed between the different groups of liver weights. On the contrary, the activity of the cathepsin B was found to be significantly higher in livers with the lowest weights, that is, lower than 650 g (*P* < 0.001).

As indicated in Table 2 by nonsignificant differences in the concentrations of their respective markers between the 4 studied groups, neither ferroptosis nor necroptosis programmed cellular death pathways were found to be differently activated in the present study.

**Figure 1:**
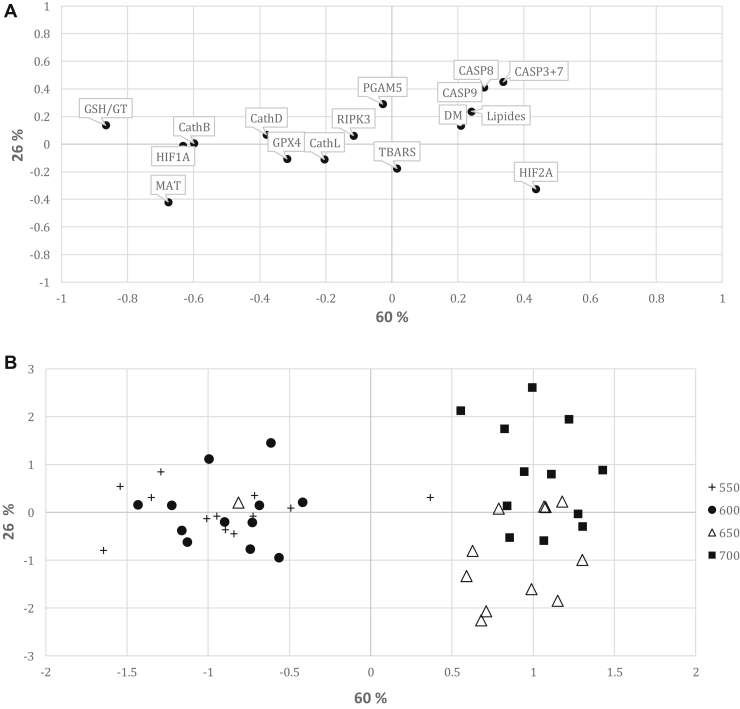
(A) Plot of the two first principal component score vectors showing relationships between the studied biochemical characteristics. (B) Plot of the two first principal component score vectors showing variability according to the 4 livers weights (LW) groups: +550 = 550 <LW < 599, • 600 = 600 <LW < 649, △ 650 = 650 <LW < 699, ▪ 700 = LW > 700 g. The percentages at the axis levels stand for the variability explained by the first (horizontally) and the second (vertically) principal components.

Figure 1 reported the results of the canonical discriminant analysis used to try to distinguish the 4 liver weights groups based on the measurements of the markers described earlier. It shows that the first (horizontal) axis is able to distinguish the 2 lightest from the 2 heaviest groups, while the second one (vertical axis) makes possible the distinction between the 2 heaviest groups but not the 2 lightest ones. The measured markers having the greatest influences in those distinctions of the groups of weights of livers are the GSH/GT ratio (relative to the oxidative status), the level of HIF1α (reporting a light hypoxia), and the activity of cathepsin B (activated in proteolysis). Those markers are of great importance to distinguish the 2 lightest from the 2 heaviest groups of livers. Of secondary importance are the activities of the caspases to separate the 2 heaviest groups of livers.

The aim of this experiment was to observe some cellular markers in the liver of ducks weighing differently but exhibiting similar levels of a nutritional steatosis as assessed by similar total lipids contents.

In ducks, the weight of the liver increases from a weight of about 80 to more than 500 g after 10 D (Théron, 2011, Rémignon et al., 2018, Bonnefont et al., 2019) of force-feeding. This is due to the development of hepatic steatosis resulting from the accumulation of triglycerides in the hepatocytes. As described by Locsmándi et al. (2007), those lipids are stored in macrovacuoles located in the cytoplasm of hepatocytes. According to Hermier et al. (1999), during the force-feeding, the lipid rate in the livers of ducks can increase from 5% to more than 50% of DM. In the present study, all livers were more or less heavy but always presented a high percentage of lipids (around 57% of DM).

Mann et al. (2017) reported that the oxidative stress is central in the pathogenesis of NASH. They suggested that for NASH, mitochondrial dysfunction is the main source of ROS and is closely related to endoplasmic reticulum stress. Both are caused by lipotoxicity, and together these factors form a cycle of progressive organelle damage resulting in sterile inflammation and apoptosis. According to Sahoo et al. (2017), GSH is the most important intracellular antioxidant. It acts as a free radical scavenger, and in combination with glutathione peroxidases, it contributes to detoxification of hydrogen peroxides by reducing ROS. Consequently, the ratio of GSH to oxidized glutathione is an indicator of cellular health, and a decrease of its value must be associated with a major cellular dysfunction (Chen et al., 2013). In mice, those authors reported that a severe depletion of hepatic GSH is associated with a massive steatosis, the death of hepatocytes, and an inflammatory infiltration. In the present study, the GSH/GT ratio decreased significantly when livers weighed more than 650 g, indicating that a high oxidative stress is present in the livers with the highest weights.

However, nonaccumulation of lipid hydroperoxides is confirmed by the lack of significance when TBARS values of the different groups are compared. It is noticeable that this lack of accumulation of lipid hydroperoxides was already suggested by Théron (2011) who reported similar levels of TBARS values in livers from force-fed duck, while Ciapaite et al. (2011) reported opposite results in studies with livers of rats exhibiting steatosis induced by a high-fat diet.

According to McGarry et al. (2018), an increase in ROS can result from a reduction of an oxygen supply efficiency which leads to a hypoxic microenvironment and mitochondrial dysfunctions. According to Suzuki et al. (2014), in mammals, altered hepatic metabolism and tissue remodeling in fatty liver disease further disrupt hepatic oxygen homeostasis resulting in severe liver hypoxia. For Lefere et al. (2016), the overexpression of HIF-1α is an essential step in the progression to NASH. Morello et al. (2018) reported that HIF-2α is overexpressed in humans or mice developing hepatic steatosis from different origins. In the present study, HIF-1α was overexpressed in the lightest fatty livers, while the highest values of HIF-2α were encountered in livers weighing more than 650 g. Those results could indicate that for fatty livers with light weight, the level of hypoxia could be considered as light, while, on the contrary, in livers exhibiting the highest weights, it might be considered as severe as demonstrated by a low level of HIF-1α and a high level of HIF-2α.

Different forms of cellular death have been explored in this experiment to check if they were activated by the increase of the weight of the liver associated with the nutritional steatosis. The major way of the programmed cellular death is apoptosis, which is mainly supported by caspases. Those proteolytic enzymes can be divided into 2 groups: initiator (isoforms 2, 9, 8, and 10) and executioner (isoforms 3, 6, and 7) caspases according to Shalini et al. (2015).

Apoptosis is one of the active pathways in patients with nonalcoholic fatty liver disease in steatohepatitis, and recently, Kanda et al. (2018) demonstrated that the activation of caspases, a key step in apoptosis, plays a role in the activation of nonalcoholic fatty liver disease/NASH. The activities of the caspases 3 and 7, markers of the irreversible phase of cells entering in apoptosis according to Shalini et al. (2015), are significantly higher for livers with the highest weights (more than 700 g). This result has already been observed by Rémignon et al. (2018). For cathepsins D and L, no difference in activities was observed between liver weight groups. However, unlike caspase 3 and 7 activities, the cathepsins B activities decrease when the liver weight rises. Rémignon et al. (2018) suggested that the rapid development of hepatic steatosis during force-feeding resulted from a reduction of the autophagic degradation of hepatocytes mediated by lysosomal proteases such as cathepsins and associated to an enhanced development of apoptosis.

Another way of the programmed cellular death could be necroptosis or ferroptosis. According to Lu et al. (2016), an increase of RIPK3 and PGAM5 concentrations is a good indicator of the activation of the necroptosis pathway. On the contrary, an increase in the level of activity of the GPX4 enzyme indicates an activation of the ferroptosis pathway (Xie et al., 2016).

Gautheron (2017) demonstrated that during the development of NASH, the necroptosis pathway can be activated. This is mainly visible through the increase of RIPK3 or PGAM5 levels in the livers of patients with NASH and by the decrease in the activity of the caspase 8, which normally inhibits necroptosis by suppressing the function of RIPK1 and RIPK3 to activate MKML. None of these variations are visible in the present study when we compare our 4 different groups of liver weights. This indicates that in steatotic livers of different weights resulting from force-feeding in ducks, the necroptosis cell death pathways are not activated.

In liver steatosis or in cases of NASH-related hepatic steatosis in diverse species including birds, the ferroptosis, a form of regulated cell death characterized by the iron-dependent accumulation of lipid hydroperoxides to lethal levels, can be observed (Conrad et al., 2018). It is mainly characterized by the inactivation of the GPX4. In the present study, we do not observe a significant variation of GPX4 activity in any group of livers differing in weights. This indicates that the ferroptosis is not present in the livers exhibiting a steatosis induced by force-feeding in ducks.

## Conclusion

Our results demonstrated that when liver weights are increased as a result of force-feeding activities, cell death pathways such as ferroptosis or necroptosis are not activated. On the contrary, for livers weighing more than 700 g, apoptosis is activated. The activation of this cell death pathway could be related to an oxidative stress consequently to a rise in the level of hypoxia in hepatocytes. However, the origin of the apoptosis occurring in heavy steatotic livers could also be more diverse. Therefore, further investigations are needed to better characterize the level of hypoxia and its consequences in livers undergoing steatosis in force-fed ducks.

## References

[bib1] Babilé R., Auvergne A., Andrade V., Héraut F., Bénard G., Bouillet-Oudot M. (1996). « Réversibilité de La Stéatose Hépatique Chez Le Canard Mulard; Proceedings of Journées de la Recherche sur les Palmipèdes à Foie Gras, Bordeaux.

[bib2] Babilé R., Auvergne A., Dubois J.P., Bénard G., Manse H. (1998). Réversibilité de la stéatose hépatique chez l’oie; Proceedings of Journées de la Recherche sur les Palmipèdes à Foie Gras, Bordeaux.

[bib3] Bénard G., Labie C. (1998). Evolution Histologique Du Foie Des Palmipèdes Au Cours Du Gavage; Proceedings of Journées de la Recherche sur les Palmipèdes à Foie Gras, Bordeaux.

[bib4] Bonnefont C.M.D., Guerra A., Théron L., Molette C., Canlet C., Fernandez X. (2014). Metabolomic study of fatty livers in ducks: Identification by 1H-NMR of metabolic markers associated with technological quality. Poult. Sci..

[bib5] Bonnefont C.M.D., Molette C., Lavigne F., Manse H., Bravo C., Lo B., Rémignon H., Arroyo J., Bouillier-Oudot M. (2019). Evolution of liver fattening and foie gras technological yield during the overfeeding period in mule duck. Poult. Sci..

[bib34] Chen Y., Dong H., Thompson D.C., Shertzer H.G., Nebert D.W., Vasiliou V. (2013). Glutathione defense mechanism in liver injury: insights from animal models. Food Chem. Toxicol..

[bib6] Ciapaite J., van den Broek N.M., te Brinke H., Nicolay K., Jeneson J.A., Houten S.M., Prompers J.J. (2011). Differential effects of short- and long-term high-fat diet feeding on hepatic fatty acid metabolism in rats. Biochim. Biophys. Acta.

[bib7] Conrad M., Kagan V.E., Bayir H., Pagnussat G.C., Head B., Traber M.G., Stockwell B.R. (2018). Regulation of lipid peroxidation and ferroptosis in diverse species. Genes Dev..

[bib8] Gautheron J. (2017). Stéatohépatite non-alcoolique et nécroptose - Les différentes facettes de RIPK3. Med. Sci. (Paris).

[bib9] Ghani M.A., Barril C., Bedgood D.R., Prenzler P.D. (2017). Measurement of antioxidant activity with the thiobarbituric acid reactive substances assay. Food Chem..

[bib10] Gradwell D.P., Rainford D.J., Gradwell D.P. (2006). Hypoxia and hyperventilatio. Ernsting's Aviation Medicine. 4th ed.

[bib11] Hermier D., Salichon M.R., Guy G., Peresson R., Lagarrigue S., Mourot J. (1999). La stéatose hépatique des palmipèdes gavés : bases métaboliques et sensibilité génétique. INRA Prod. Anim..

[bib12] Hermier D., Guy G., Guillaumin S., Davail S., Amdré J.M., Hoo-Paris R. (2003). Differential channelling of liver lipids in relation to susceptibility to hepatic steatosis in two species of ducks. Comp. Biochem. Physiol. B Biochem. Mol. Biol..

[bib13] Kanda T., Matsuoka S., Yamazaki M., Shibata T., Nirei K., Takahashi H., Kaneko T., Fujisawa M., Higuchi T., Nakamura H., Matsumoto N., Yamagami H., Ogawa M., Imazu H., Kuroda K., Moriyama M. (2018). Apoptosis and non-alcoholic fatty liver diseases. World J. Gastroenterol..

[bib14] (2018). Comité Interprofessionnel des Palmipèdes à Foie Gras (CIFOG).

[bib15] Lefere S., Van Steenkiste C., Verhelst X., Van Vlierberghe H., Devisscher L., Geerts A. (2016). Hypoxia-regulated mechanisms in the pathogenesis of obesity and non-alcoholic fatty liver disease. Cell. Mol. Life Sci..

[bib16] Locsmándi L., Hegedüs G., Andrássy-Baka G., Bogenfürst F., Romvári R. (2007). Following the goose liver development by means of cross-sectional digital imaging, liver histology and blood biochemical parameters. Acta Biol. Hung..

[bib17] Lu W., Sun J., Yoon J.S., Zhang Y., Zheng L., Murphy E., Mattson M.P., Lenardo M.J. (2016). Mitochondrial protein PGAM5 Regulates Mitophagic Protection against cell necroptosis. PLoS One.

[bib18] Lynch S.M., Frei B. (1993). Mechanisms of copper- and iron-dependent oxidative modification of human low density lipoprotein. J. Lipid Res..

[bib19] Machado M.V., Diehl A.M. (2016). Pathogenesis of nonalcoholic steatohepatitis. Gastroenterology.

[bib20] Mann J.P., Raponi M., Nobili V. (2017). Clinical implications of understanding the association between oxidative stress and pediatric NAFLD. Expert Rev. Gastroenterol. Hepatol..

[bib21] Marie-Etancelin C., Vitezica Z.G., Bonnal L., Fernandez X., Bastianelli D. (2014). Selecting the quality of mule duck fatty liver based on near-infrared spectroscopy. Genet. Sel Evol..

[bib22] McGarry T., Biniecka M., Veale D.J., Fearon U. (2018). Hypoxia, oxidative stress and inflammation. Free Radic. Biol. Med..

[bib23] Morello E., Sutti S., Foglia B., Novo E., Cannito S., Bocca C., Rajsky M., Bruzzì S., Abate M.L., Rosso C., Bozzola C., David E., Bugianesi E., Albano E., Parola M. (2018). Hypoxia-inducible factor 2α drives nonalcoholic fatty liver progression by triggering hepatocyte release of histidine-rich glycoprotein. Hepatology.

[bib24] Rankin E.B., Rha J., Selak M.A., Unger T.L., Keith B., Liu Q., Haase V.H. (2009). Hypoxia-Inducible factor 2 Regulates hepatic lipid metabolism. Mol. Cell Biol..

[bib25] Rémignon H., Ben Haj Yahia R., Marty-Gasset N., Wilkesman J. (2018). Apoptosis during the development of the hepatic steatosis in force-fed ducks and cooking yield implications. Poul. Sci..

[bib26] Sahoo S., Awasthi J.P., Sunkar R., Panda S.K., Sunkar R. (2017). Determining glutathione levels in Plants. Plant Stress Tolerance.

[bib27] Shalini S., Dorstyn L., Dawar S., Kumar S. (2015). Old, new and emerging functions of caspases. Cell Death Differ.

[bib28] Souci S., Fachmann W., Kraut H., Scherz H., Senser F. (2000). Food Composition and Nutrition Tables.

[bib29] Suzuki T., Shinjo S., Arai T., Kanai M., Goda N. (2014). Hypoxia and fatty liver. World J. Gastroenterol..

[bib30] Théron L. (2011). Déterminisme biologique de la variabilité de la fonte lipidique à la cuisson du foie gras de canard (*Biological determinism of the variability of fat loss during cooking of duck 'foie gras'*). http://ethesis.inp-toulouse.fr/archive/00001683/.

[bib31] Tsurusaki S., Tsuchiya Y., Koumura T., Nakasone M., Sakamoto T., Matsuoka M., Imai H., Yuet-Yin Kok C., Okochi H., Nakano H., Miyajima A., Tanaka M. (2019). Hepatic ferroptosis plays an important role as the trigger for initiating inflammation in nonalcoholic steatohepatitis. Cell Death Dis..

[bib32] UE. (2008). RÈGLEMENT (CE) N o 543/2008 DE LA COMMISSION du 16 juin 2008. https://eur-lex.europa.eu/legal-content/FR/TXT/HTML/?uri=CELEX:32008R0543&from=FR.

[bib33] Xie Y., Hou W., Song X., Yu Y., Huang J., Sun X., Kang R., Tang D. (2016). Ferroptosis: process and function. Cell Death Differ..

